# Plant genotype, microbial recruitment and nutritional security

**DOI:** 10.3389/fpls.2015.00608

**Published:** 2015-08-07

**Authors:** Jai S. Patel, Akanksha Singh, Harikesh B. Singh, Birinchi K. Sarma

**Affiliations:** ^1^Department of Botany, Banaras Hindu UniversityVaranasi, India; ^2^Department of Mycology and Plant Pathology, Institute of Agricultural Sciences, Banaras Hindu UniversityVaranasi, India

**Keywords:** plant genotype, rhizosphere microbe, nutritional security, PGPR, mycorrhiza

## Abstract

Agricultural food products with high nutritional value should always be preferred over food products with low nutritional value. Efforts are being made to increase nutritional value of food by incorporating dietary supplements to the food products. The same is more desirous if the nutritional value of food is increased under natural environmental conditions especially in agricultural farms. Fragmented researches have demonstrated possibilities in achieving the same. The rhizosphere is vital in this regard for not only health and nutritional status of plants but also for the microorganisms colonizing the rhizosphere. Remarkably robust composition of plant microbiome with respect to other soil environments clearly suggests the role of a plant host in discriminating its colonizers (Zancarini et al., 2012). A large number of biotic and abiotic factors are believed to manipulate the microbial communities in the rhizosphere. However, plant genotype has proven to be the key in giving the final shape of the rhizosphere microbiome (Berendsen et al., 2012; Marques et al., 2014).

Plants are reported to release up to 40% of their photosynthetic products into soil and thereby provide a strong and stable foundation for building up of the rhizosphere microbiome (Bais et al., 2006). Root exudates differ depending on the plant species and the developmental stages of plant (Jaeger et al., 1999). Thus, the host plant is believed to influence the relative plenitude of indigenous rhizobacteria. Berg et al. (2006) further demonstrated that alteration in composition of bacterial and fungal species in the rhizosphere is dependent on the plant species. Similarly, under certain stressful conditions plants release organic acids such as citric, succinic, and malic acids more in the root exudates and release of such acids helps the host plants to recruit certain microbial species. Recruitment of microbial species by such mechanisms is known to relieve stresses in vegetable crops such as tomato (*Lycopersicon esculentum*), cucumber (*Cucumis sativus*), and sweet pepper (*Capsicum annum*; Kamilova et al., 2006). Further it is proved that different genotypes of the same plant species may also have significant impacts on selecting “rhizospheric partners” through production of diverse root exudates. Additionally, recent findings suggest that differences in even a single gene between plant genotypes can have a noteworthy influence on the rhizosphere microbiome. Microbial community residing on the roots of transgenic *Arabidopsis* is altered following production of a single exogenous product glucosinolate (Bressan et al., 2009). Similarly, Cotta et al. (2014) demonstrated that abundances of ammonia-oxidizing bacterial and archaeal communities in the rhizosphere is changed in transgenic maize genotypes expressing different Bt toxin genes Cry1Ab and Cry1F.

As described above, the benefits are not uni-directional and plants are also benefitted by microbial colonization. The role of microbes in enhancing plant’s capacity to resist biotic and/or abiotic stresses through production of a range of compounds is successfully demonstrated. The compounds include various secondary metabolites which are well known for their antioxidant activities and other health beneficial results. In addition to increasing plant growth and concentrations of defense metabolites some recent studies also showed that nutritional values are also enhanced in plants inoculated with beneficial microbes. Singh et al. (2014) demonstrated that seeds inoculated with selected rhizosphere compatible microbes such as fluorescent *Pseudomonas*, *Trichoderma*, and *Mesorhizobium* species increased the quantities of antioxidant phenolics to several folds in the edible parts of chickpea. Similarly, Jain et al. (2014) also demonstrated that microbial mixtures can enhance nutritional value of pea seeds. High intake of plant products rich in phenols and antioxidants can reduce risks of many chronic diseases such as atherosclerosis, cancer, diabetes, Alzheimer’s, Parkinson’s diseases, etc. In another report Sahni et al. (2008) showed that seed bacterization with a fluorescent *Pseudomonas* PUR46 increased the availability and uptake of minerals like P, Mn, and Fe in chickpea. Biofortification of crop plants with other minerals such as Zn is also possible through utilization of rhizospheric Zn-mobilizing bacteria (Hafeez et al., 2013). Moreover, endophytic and associative symbionts are being utilized for supplement of N in non-leguminous crops particularly in cereals (Santi et al., 2013). Application of such microbes also promote crop growth and reduce diseases significantly by both direct and indirect mechanisms. Similarly, arbuscular mycorrhiza (AM) increased the amounts of nutritionally important elements like copper (Cu) and iron (Fe) in lettuce (Baslam et al., 2011) and total antioxidants and minerals in onion bulb (Albrechtova et al., 2012). In another study, tomato plants inoculated with AM fungi showed significantly higher concentrations of glucose and malate in the fruits (Copetta et al., 2011). Further, an interesting study showed that inoculation of *Rhizobium* had a positive effect on a non-leguminous crop viz., *C. annum* (Silva et al., 2014). The *Rhizobium* species enhanced not only fruit ripening significantly but also increased the concentrations of some primary and secondary metabolites having high nutritional values.

Further, it is known that defense responses in plants are induced via the jasmonate pathway involving jasmonic acid when primed with beneficial microbial species. Such induced defense signaling can also have an indirect effect on increasing the concentrations of compounds such as resveratrol in *Vitis vinifera* (Ahuja et al., 2012), glucosinolate in cruciferous vegetables (Grubb and Abel, 2006), anthocyanins and antioxidant compounds in fruits and vegetables (Wang and Zheng, 2005), etc., which are known to have medicinal values. Many of the hydrophilic antioxidants (anthocyanins, flavonols, isoflavonoids, or chlorogenic acid) and the lipophilic antioxidants (lycopene or vitamin E) were also accumulated in higher amounts upon priming the plants with rhzosphere bacteria and fungi (Jain et al., 2014). Similarly, de Santiago et al. (2013) showed that Fe acquisition in wheat, white lupin and cucumber plants increased when *Trichoderma asperellum* strain T34 was inoculated in the calcareous media. Protein content in chickpea was increased by 16% and in pea by 8% following inoculation with *Mesorhizobium* sp. RC3 and *Rhizobium* sp. RP5, respectively (Wani et al., 2008a,b). Thus, taking the leads from the above studies, it can be concluded that priming the plants with beneficial microbes holds great promise in improving nutritional values of food especially in the developing countries where nutritional security is a major concern.

From the findings listed above it is evident that plant genotype and species affects the process of microbial recruitment in the rhizosphere of a plant species. It is achieved through release of large quantities of organic compounds as root exudates and the root exudate profiles vary with plant genotype and species (Bakker et al., 2013). Subsequently these microbes in the rhizosphere play a significant role in promoting plant growth, relieving biotic and abiotic stresses and increasing nutritional value of the edible plant parts. However, systematic efforts were not made to focus on utilization of plant genotypes targeting microbial recruitment in the rhizosphere to get the desired benefits from such recruitments. Unlike extensive research that led to identification of good biological control agents for management of agricultural pests and pathogens, lesser efforts were made to identify microbes that have capabilities to increase nutritional value of consumable agricultural produces. It is therefore important to reorient researches toward attaining nutritional security through use of selective plant genotypes. Such selectively utilized plant genotypes can facilitate recruitment of desirable microbial species in the rhizosphere and thereby enhance nutritional value of agricultural produces. Once the host’s mechanism(s) to recruit desired microbial species in the rhizosphere is established, other genotypes can also be engineered to facilitate the process either through conventional breeding programs or through biotechnological interventions. Time has come for an integrated effort from microbiologists, plant breeders, biotechnologists, and nutritionists to join hands for the noble cause of ensuring nutritional security to all.

## Conflict of Interest Statement

The authors declare that the research was conducted in the absence of any commercial or financial relationships that could be construed as a potential conflict of interest.
